# Exploring Multiple Dimensions of Access to and Preferences for Telehealth Use

**DOI:** 10.1089/tmr.2023.0049

**Published:** 2023-12-08

**Authors:** Kristin Pullyblank, Melissa Scribani, Nicole Krupa, Amanda Chapman, Megan Kern, Wendy Brunner

**Affiliations:** ^1^ Center for Rural Community Health, Bassett Research Institute, Bassett Medical Center, Cooperstown, New York, USA.; ^2^ Center for Biostatistics, Bassett Research Institute, Bassett Medical Center, Cooperstown, New York, USA.

**Keywords:** telehealth, telemedicine, health equity, access, patient preference

## Abstract

**Introduction:**

During the pandemic, telehealth became critically important in care provision. Yet, research exposed the inequities facing various groups of people in terms of accessing telehealth. The purpose of this analysis was to examine the various dimensions of access that impact a person's ability to use and preference for telehealth.

**Methods:**

We used a mixed-methods approach framed by Levesque's Access to Health care model. In August, 2021, a stratified random sample of 500 patients of an integrated rural health care network was invited to participate in a survey designed to capture familiarity with, use of, and preference for digital technologies in general as well as with telehealth. In addition, key informant interviews were conducted between January 2022 and June 2022.

**Results:**

Patients' willingness to use telehealth was influenced by multiple dimensions of access, including approachability of the resource, acceptability, availability, affordability, and appropriateness. Clinician beliefs and attitudes as well as health care system policies affected how a patient perceived, sought, reached, and engaged with telehealth.

**Conclusions:**

Access is a dynamic, multifaceted concept that is influenced by individual-, organization-, and systemic-level factors. Looking beyond patient determinants and examining different dimensions of access is important to better facilitate implementation and sustainment of telehealth.

## Introduction

Health care systems have rapidly implemented digital health technologies as a viable solution to the barriers that affect patients' access to care, including an unprecedented health care workforce shortage, transportation difficulties, and scheduling conflicts.^1–3^ While the pandemic was a catalyst for dramatic increases in telehealth use, the extent of telehealth adoption in the long-term remains unclear. Recent data from hospital systems suggest telehealth use is leveling out at 10–20% of total volume.^4^ One of the concerns facing widespread adoption is equitable access to these services. Before the pandemic, most studies on telehealth use and acceptance were small-scale pilot projects.^5^ Research since the start of the pandemic has addressed changes in telehealth use,^6–8^ characteristics of users,^9,10^ and barriers and facilitators to use.^11–14^ Many studies have focused on disparities in use,^5,15–19^ which is ostensibly a function of “access.”

The concept of access is often reduced to characteristics that influence the use of services.^20^ This oversimplification fails to provide an appropriate level of context that is required to develop effective solutions. For example, while it is known that older people are less likely to use telehealth,^10,21^ it is impossible to address the issue until we can understand the basis of that disparity. Is it because of inability to use the technology, lack of trust in the technology, or a preference for human contact during medical appointments? Similarly, most literature since the start of the pandemic has noted that rural residents are not adopting telehealth as quickly as their urban counterparts,^8,18^ yet it is unclear if this is due to lack of internet coverage, cultural attitudes, or something else. If we want to ensure that all patients have equitable access to telehealth, we have to truly understand the factors that are causing these disparities.

**FIG. 1.**
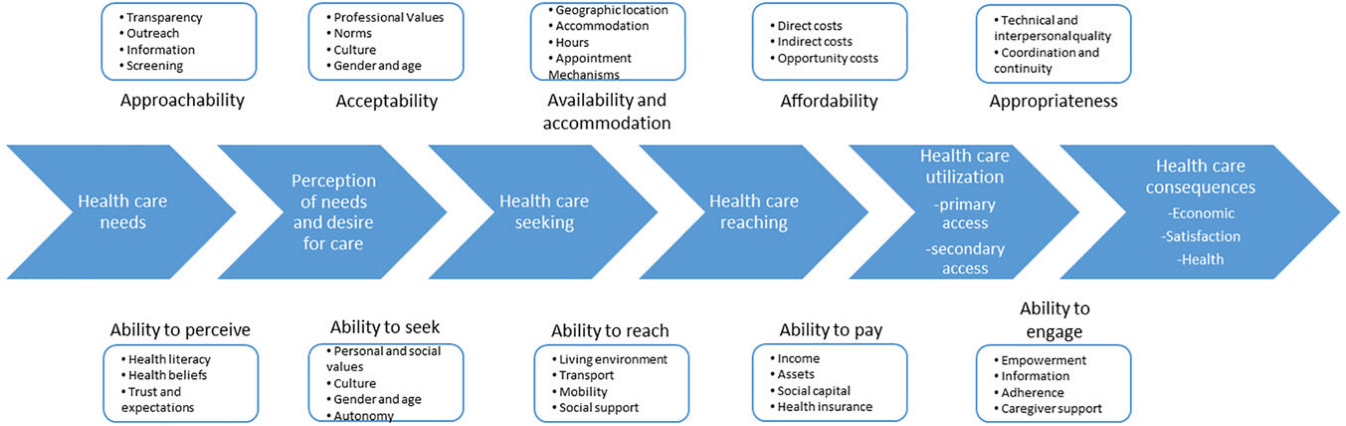
Access to health care model (adapted from Levesque et al.^20^).

Levesque et al. conceptualized multiple dimensions of access (Fig. 1) originating from both the supply side (e.g., health care systems, organizations) and demand side (e.g., individuals, families, communities), across different processes in which access is realized (e.g., health care needs, health care seeking, health care reaching).^20^ The dimensions include approachability (ability to perceive), acceptability (ability to seek), availability and accommodation (ability to reach), affordability (ability to pay), and appropriateness (ability to engage).

The purpose of this mixed-methods analysis was to explore the specific dimensions of access that impact a person's ability to successfully use telehealth. Uncovering this information provides insight into specific strategies health care systems can employ to increase utilization.

## Methods

The analysis was part of a larger telehealth equity study framed by Crawford and Serhal's digital health equity model.^15^ This analysis was designed using a mixed-methods approach due to the complex nature of understanding health behavior and access to care when studying emerging technologies. Specifically, we employed a sequential explanatory design, which allowed our qualitative data to illuminate our quantitative findings.^22^ We used data from a cross-sectional survey disseminated in the summer of 2021 and key informant interviews conducted between January 2022 and July 2022. The quantitative data from the survey allows us to make some inferences about population-level trends in the region regarding telehealth use and preferences, while the qualitative interviews provide important contextual data to help interpret the trends that we are observing.

The study was approved by the hospital's Institutional Review Board (Projects 1850342 and 1774409). All participants provided informed consent. This analysis is organized using Levesque's conceptual framework of health care access.^20^ Analysis of survey and interview data will be independently discussed in the results, with the integration occurring in the discussion.^22^

### Setting

The study was conducted within our integrated health care network, located in rural central upstate New York. Nearly 90% of residents are white, non-Hispanic. Approximately 21% of the population is 65 years and older (compared with the New York state average of 17.5%) and 13% of residents do not have broadband internet access. Poverty rates for the region are similar to New York State, at 13.7% (U.S. Census).

### Key terms

For the purposes of this study, *digital health technologies* refer to any digital tools that support health. *Telehealth* refers to a medical appointment between a clinician and a patient that occurs remotely (i.e., patient is not in clinic/doctor's office). On the survey, this was also called *remote appointments. Videoconferencing* refers to use of a web-based video platform for any purpose, including for telehealth appointments. *Audio-only telehealth* are remote appointments conducted through phone (no video screen). An *e-visit* is an online, self-guided health assessment, triage, and treatment tool.

### Survey

The survey instrument (see Supplementary Data) included items assessing familiarity with and use of computers, smartphones, the internet, and videoconferencing platforms; preferences for accessing the internet outside of the home; use of telehealth before and during the pandemic and associated facilitators and barriers; and preferences for visits with clinicians (e.g., through telehealth or in-person). The survey also included a variety of sociodemographic questions, including age, race, gender, marital status, education, health status, disability status, and insurance status. Rurality was defined by using Rural-Urban Commuting Area (RUCA) codes, which were derived from the respondent's ZIP code.

Survey items were developed based on a review of the literature as well as through consultation with experts in digital technology use, health behavior, epidemiology, and biostatistics. The survey was piloted with 10 individuals before dissemination. A random sample consisting of 500 adult (18 years or older) patients who had at least one office visit or telemedicine visit within our health care network between May 1, 2020 and July 15, 2021, was community dwelling, and lived within the network service area was invited to participate.

The sample was stratified based on access/no access to the patient portal (a proxy for exposure to digital health technologies) with 250 individuals in each stratum. This stratification was deemed necessary as we wanted to reach a large-enough sample who had not previously used telehealth. At the time of survey dissemination, 63% of the adult patient population within the health care network had access to the patient portal. Surveys were sent through the patient portal to those who had access; and mailed through U.S. Postal Service for those who did not. Follow-up surveys were mailed through U.S. Postal Service to all nonresponders between 2 and 4 weeks after the initial surveys were sent.

### Survey analysis

We used descriptive statistics to summarize group demographic characteristics, telehealth use characteristics, and reported barriers and benefits to telehealth use. To determine predictors of preferences for telehealth versus in-person appointments, we conducted bivariate analyses using the chi-square test for categorical variables and the *t*-test for age as a continuous variable. To identify independently associated factors with visit preference, we constructed a multivariable logistic regression equation predicting odds of preference for in-person visits as the outcome. Variables that showed a significant association with visit preference on bivariate testing (*p* < 0.05) were retained in the multivariable model. In addition, variables shown in prior studies to be significantly associated with telehealth utilization were included.

The final logistic model included the following covariates: previous exposure to telehealth (yes or no), previous exposure to videoconferencing (yes or no), gender (male or female), age (continuous), financial distress (yes or no), marital status (married/partnered or not), educational attainment (at least some college education or not), disability status (yes or no), and self-rated health (good/very good/excellent vs. fair/poor).^21,23–25^

### Qualitative interviews and analysis

Two members of the research team (K.P., A.C.) conducted a series of interviews with ten key informants, including clinicians (*n* = 2), schedulers (*n* = 2), patients (*n* = 3), the virtual health department (*n* = 1), patient experience department (*n* = 1), and a member of the patient family advisory board (*n* = 1). Informants were recruited through direct outreach to the virtual health and patient experience departments. Key informants in these departments then suggested possible clinician, support staff, and patient informants.

Using a semistructured interview guide based on the digital health equity model, key informants were asked about digital health literacy, quality of care, barriers and facilitators to telehealth use, and integration of digital health resources into the community infrastructure during the first interview. In the second round of interviews, the research team shared the survey data results and asked participants their impressions regarding the findings. All interviews were transcribed and uploaded into NVivo. The general analytic approach was inductive thematic analysis, where text was coded inductively to identify themes from the data.^26^

Following Braun and Clark's six step process,^27^ the first author developed initial codes from the first round of interviews and then began to explore themes in the data. An iterative process of constructing, diagramming, and refining themes, and then returning to the data, ensued. Several strategies were used to ensure trustworthiness.^26^ First, the entire research team debriefed regularly and maintained an audit trail throughout the analytic process. In addition, the initial themes were brought back to the key informants during second round interviews for participant feedback. This process resulted in further refinement of themes. All interviews were conducted over Zoom by study members experienced in qualitative methods.

## Results

### Survey

Of the 206 individuals who responded to the survey (41.2%), 110 had access to the patient portal. The median age was 63 years, 61% were female, and 96% were white. Full demographics can be found in Table 1. Nearly 85% reported having internet in their home, and 73% reported being able to use the internet independently. Of the twenty-nine respondents reporting no internet in the home, reasons included prohibitive cost (23.3%), did not want or need (30.0%), did not know how to use the internet (15.0%), did not know how to use computer (23.3%), and internet not available in the area (8.3%).

**Table 1.** Characteristics of Survey Sample

Variable	***n*** (%)
Age	
Median age (SD)	63 (17.09)
<65	110 (53.4)
65+	96 (46.6)
Race	
White	191 (96.0)
Non-White	8 (4.0)
Ethnicity	
Hispanic	199 (99.0)
Non-Hispanic	2 (1.0)
Gender	
Female	125 (60.7)
Male	81 (39.3)
Education	
High school or less	80 (39.6)
At least some college	122 (60.4)
Marital status	
Married/partnered	117 (58.2)
Not married/partnered	84 (41.8)
Employment status	
Working	73 (36.0)
Not working	130 (64.0)
Rurality (RUCA)	
Metro/micro (1–6)	101 (49.3)
Rural (7–10)	104 (50.7)
Medicare status	
Yes	97 (48.0)
No	105 (52.0)
Medicaid status	
Yes	50 (24.8)
No	152 (75.2)
Household income	
<$30,000	68 (37.4)
$30,000+	114 (62.6)
General health	
Excellent/very good/good	156 (77.6)
Fair/poor	45 (22.4)
Disability	
Yes	43 (21.3)
No	159 (78.7)

SD, standard deviation; RUCA, Rural-Urban Commuting Area.

### Appointment type use and preferences

Video or audio-only telehealth had been used by 42.0% of survey respondents at the time the survey was disseminated and 3.5% of respondents reported using an e-visit accessed through the patient portal. However, nearly three-quarters of respondents (73.6%) responded they would prefer an in-person visit, even if the appointment were appropriate for a telehealth visit.

Bivariate analyses of appointment preference versus various demographic determinants indicated that preference for remote versus in-person visits was associated with having prior experience with telehealth and younger age (Table 2). There was no association in preferences based on gender, income, self-reported health, education, disability status, marital status, or rurality.

**Table 2.** Association Between Visit Type Preference and Prior Experience with Telehealth and Age Group

	Remote visit using computer with video ***n*** (%)	Remote, audio only ***n*** (%)	In person ***n*** (%)	***p***
Prior experience with telehealth (audio or videoconference)	19 (24.1)	12 (15.2)	48 (60.8)	0.001
No prior experience with telehealth (audio or videoconference)	15 (12.8)	5 (4.3)	97 (82.9)	
<65 years old	30 (28.8)	9 (8.7)	65 (62.5)	<0.001
65 or older	4 (4.3)	9 (9.7)	80 (86.0)	

Significant independent predictors of preference for in-person visits included no prior exposure to telehealth, being older, being male, and having at least some college education (Table 3).

**Table 3.** Multiple Logistic Regression Model Predicting Preference for In-Person Appointments

	***B***	SE	Sig	OR	95% CI for OR	
					Lower	Upper
No previous exposure to telehealth	2.08	0.49	<0.001	7.94	3.07	20.54
Gender: male	1.23	0.52	0.019	3.41	1.23	9.44
Age	0.06	0.01	<0.001	1.06	1.03	1.09
Education: at least some college	1.17	0.49	0.016	3.21	1.24	8.29
Not married/partnered	0.51	0.48	0.28	1.67	0.65	4.28
Endorsed disability	−0.62	0.58	0.28	0.54	0.17	1.67
Health: fair or poor	.33	0.55	0.55	1.38	0.48	4.03

OR, odds ratio; SE, standard error.

### Barriers and benefits of remote appointments

Participants were asked to self-report the benefits and barriers to remote doctor's appointments and other health-related programs. Participants were allowed to select all that apply.

Not having to leave home, not having to drive, not having to wait in the waiting areas, and taking less time than traditional visits were seen as key benefits to telehealth appointments (Table 4). Sixty-four respondents (33.7%) reported no benefits to remote appointments.

**Table 4.** Reported Benefits and Barriers of Remote Appointments

	%
Benefits	
Don't have to leave home	55.3
Don't have to drive	43.2
Don't have to wait	40.0
Takes less time	39.5
Don't have to be around others	30.0
More flexible appointment times	28.4
Don't have to miss work	24.7
More flexible provider choice	11.1
Barriers	
Not in person	55.8
Have to share information over the internet	20.6
I don't want to use the technology	20.1
Technology not reliable	16.1
I'm not able to use the technology	15.6

Over half of respondents believed “not being in person” was a barrier to telehealth appointments. Forty-one respondents (20.6%) reported having to share information over the internet as a barrier to remote appointments. Forty-five respondents (22.6%) reported there were no barriers to remote appointments. Chi-square analysis indicated significant associations between a preference for in-person versus remote visits and the following barriers: not wanting to use the technology (*p* = 0.026) and not being in person (*p* < 0.001). Individuals who reported no barriers to remote appointments were more likely to prefer remote visits with their clinician than those who reported at least one barrier (*p* = 0.010). Similarly, those who reported no benefits to remote appointments were significantly more likely to prefer an in-person appointment (*p* < 0.001).

### Qualitative themes

Based on the 10 key informant interviews, three main themes were developed. These were: desiring convenient, quality care; facing challenges with implementation; and needing education to trust telehealth.

#### Desiring convenient, quality care

Both patient and clinician informants wanted to be engaged with quality health care. Telehealth offered dimensions of convenience and flexibility. For patients, the opportunity to engage in telehealth meant they would not have to drive long distances to see a specialist. On-demand video appointments and e-visits gave busy individuals the flexibility to seek assessment and treatment for an acute need.

For clinicians, telehealth visits provided additional context of the patient's world by literally allowing the clinician to view the home environment. In addition, telehealth was seen as a useful mode to provide reassurance to concerned patients who overused services. For example, a clinician recalled a story where her patient had so many more in-person convenient care/urgent care visits when a nurse or clinician was not available to reassure her on the phone at the front end.

While nearly all key informants recognized the convenience and flexibility of digital health technologies, several informants also expressed concern about the quality of care that would be offered through telehealth and voiced that telehealth would only be acceptable in certain situations. The inability to conduct a physical exam in a fully remote environment was a particularly salient point for patients and clinicians. “You can't listen to their lungs. Can't get to touch. I'm a nurse to the core, and sometimes, just touching their arm, or holding their hand or putting your hand on their back or giving them a hug when things are really rough.” Another clinician believed that clinicians “felt like maybe we were somehow short-changing the patient. That we're trying to cut corners.”

Outpatient behavioral health appointments were seen as one area where telehealth was advantageous because physical exams are typically not required. As one medical office assistant said, “Patients like the option of just sitting at home and being able to do a video visit.”

Follow-up appointments were also seen as appropriate for telehealth. “In my department, we have a duty to follow up with our patients on side effects and toxicities and things like that. And for patients that had kind of a predictable low risk of toxicities…we were just finding that we were bringing people into the clinic to see us for basically us to talk to them as much as we are talking to each other right now, and to just confirm that there's nothing wrong. …We used up a lot of time for the clinics and the patients had no doubt driven way longer than they needed to, to see me to tell them ‘you're right, there's nothing wrong with you’.”

#### Facing challenges with implementation

Individuals experienced difficulty accessing the health care system in general, and key informants saw telehealth as a strategy to help improve access. However, the rapidity in which telehealth was rolled out during the pandemic resulted in inconsistent processes and procedures. For example, there was very little technical support when technological issues arose. Clinicians would often start a videoconference call only to revert to a traditional phone call because the technology was not working on the clinician's end. Depending on whether a patient had access to the patient portal, the technology used for the appointment could also vary. Key informants believed many individuals lacked access to the internet or smartphones and therefore could only participate via landline. As one patient mentioned, “the concept is fantastic but the tools aren't there.”

Transitions require changes in workflows, which creates resistance. The interviews clarified that significant changes in processes had to occur to implement a telehealth appointment. The schedulers have to schedule differently, the “rooming” process is different, and the service is billed differently. As one clinician mentioned, “people are used to doing things the way they are used to doing them.” Patients may also have been resistant because of established rituals around doctor's appointments. For older patients particularly, going to the doctor's office was seen as an event. In addition to going to the doctor, the patient may be able to go out to lunch or run other errands.

#### Needing education to trust telehealth

Multiple key informants mentioned that education was needed to increase confidence in using telehealth. For example, an older patient mentioned, “I haven't done it, so I don't know whether I have to take my own blood pressure, my own temperature, you know…I don't really know what all is involved.” It is unclear whether this lack of exposure was a function of their clinician's choice, the nature of the appointment, the perception of the clinician on the patient's ability to use telehealth, or other factors. Another concern among patients was the uncertainty around whether health appointments over the internet could be safe from cyber attacks. For example, patients were wary of opening any email links, not knowing if it was legitimate, and were often hesitant to share their information over the internet.

Some key informants believed that hosting educational forums would be helpful so that patients could learn more about the technology that is involved in a telehealth visit. Others mentioned that learning through trusted messengers (friends or family) would instill confidence in the technology.

Key informants agreed that the cornerstone of quality health care was to have a trusting relationship between clinician and patient. Several key informants mentioned that once patients were exposed to telehealth, they tended to like it. Having a trusting relationship with a clinician before a telehealth visit helped facilitate that transition to using telehealth. As one clinician said, “A lot of people are willing to [engage in telehealth] if they're kind of coached that it's the right thing to do.”

## Discussion

The purpose of this analysis was to understand why patients are or are not deciding to use telehealth within a rural integrated health care network. The discussion will integrate the quantitative and qualitative findings using Levesque et al.'s accessibility model.^20^

### Approachability and acceptability: the ability to perceive and seek

Approachability and acceptability connect to the ideas of a person realizing that receiving care through telehealth is a possibility and would be helpful for them. We found that while many individuals were aware of telehealth, questions remained about whether they would find it helpful for their own needs. Over half of survey respondents reported a barrier to remote appointments was that they were not in person, and three-quarters of survey respondents stated they would prefer an in-person appointment. Age was a factor among patients in determining preferences for telehealth with those over 65 being more likely to prefer an in-person appointment (86% vs. 62.5%).

In their study on attitudes toward telemedicine among urban and rural patients in Alabama, Tipre et al. found that 43.6% of their survey respondents would prefer to have an in-person visit compared with 24.8% by phone and 31.9% through a virtual visit.^28^ However, the average age of that study population was only 43, compared with our average age of 61. Research has consistently found that older individuals are less likely to use telehealth.^9,23,29^

Acceptability of digital health technologies may be related to rural culture. In addition to preferring in-person visits, a fifth of survey respondents reported not wanting to share their information over the internet (20.6%). Similarly, among the 29 individuals who reported not having home-based internet, 62% stated it was because they felt like they did not want or need it. A key informant mentioned that for some patients, visiting the doctor is ritualized, in that it offers a reason to “go out” and socialize.

These responses align with the rural constructs of valuing close community relationships and being wary of outside innovation disrupting the social and economic fabric of the community.^5,30^ Warr et al. suggested that to increase acceptability of telehealth services, communities must be involved in the design, implementation, and evaluation of telehealth technologies.^5^

Our study demonstrates that those with prior telehealth experience are more likely to prefer telehealth (39% vs. 17%), which echoes previous research. For example, Holtz et al. found that nontelehealth users were more likely to believe they would get better care in person, that the provider would not be as caring over telehealth, and that continuity of care would be jeopardized versus those who had experienced telehealth.^12^ Tipre et al. found that experience with virtual visits as well as younger age were independent predictors of preferring telehealth.^28^ Thus, the challenge is in initiating that first telehealth visit, allowing individuals to perceive that telehealth would be beneficial to them. Therefore, the provision of education and information is essential to increase digital health literacy, including comfort with the technology.^31^ In fact, a review of telehealth implementation in primary care indicated that a major facilitator to telehealth implementation was the provision of technological and skill-based training for both clinicians and patients.^13^

### Availability and accommodation and ability to reach

There was a general perception among survey respondents and key informants that using telehealth provided more flexibility. Over half of respondents (55.3%) perceived not having to leave home as a benefit to remote appointments. Over a quarter (28.4%) believed that remote appointments offered more flexible appointment times and 24.7% saw not having to miss work as a benefit to remote appointments. In addition, telehealth was seen as a way to engage with specialists without having to travel long distances. The accommodations that telehealth offers have been well described in the literature.^2,12,32^

However, some survey respondents and several key informants indicated that while they would like to use telehealth, they did not have the technical capabilities to engage with video telehealth. Twenty-eight percent of survey respondents reported being unable to use the internet independently. The key informants also raised other concerns about accommodation, including reaching those who need interpreters or who have sensory issues. The barriers to availability and accommodation for people to reach health care services with telehealth has also been discussed in the literature.^5,14^

Finally, even among those who believe they would like to use telehealth, the service was never offered to them. Again, it is unclear whether the lack of telehealth availability was due to clinician preference, whether the visit would not be appropriate for telehealth, or another reason.

### Affordability and ability to pay

During the public health emergency, telehealth appointments were being reimbursed at the same rate as in-person appointments. There has been much discussion about what telehealth reimbursement will look like as we move out of the Public Health Emergency.^33^ While the Consolidated Appropriations Act extended some of the provisions for Medicare recipients, including the use of audio-only telehealth in certain circumstances, as well as allowing the patient's home to be a point of origin,^33^ these are set to expire at the end of 2024. It is unclear how reimbursement rates for telehealth will compare to in-person rates in the future. Health care systems need to see a return on investment for telehealth services to continue providing them. Therefore, access is also driven by organizational priorities as well as priorities at the state and federal levels.^34^

At the individual level, concerns about cost for internet were mentioned by 14 of the 29 survey respondents who reported not having home internet. In contrast, key informants readily identified telehealth as a cost-effective solution to in-person appointments. The time and money saved by not having to travel, particularly in bad weather, was seen as a very real advantage for patients and is addressed in previous research.^2^ In addition, key informants mentioned numerous ways in which a person can connect to internet for a videoconferencing appointment without having internet in the home (e.g., connect through cell phone service).

### Appropriateness and ability to engage

One of the major challenges in expanding telehealth access is determining when and how it is appropriate to use telehealth to provide and receive care. At the time of our study, there was considerable uncertainty as to whether telehealth would be able to provide the same level of care as in-person appointments. While our survey questions did not specifically ask about how telehealth affects perceived quality of care, our key informants mentioned these concerns. Before the pandemic, most telehealth studies were smaller pilot studies and the evidence regarding telehealth's effectiveness was unclear.^35^ The uncertainly around effectiveness could be contributing to low adoption rates.

Furthermore, key informants mentioned the lack of standard protocols about when it is appropriate to use telehealth leaving the decision to the discretion of the department or individual clinician. Different academic and professional groups are currently attempting to delineate protocols for when telehealth is appropriate.^36–39^

In the meantime, patients need to be able to trust their individual clinician that telehealth is appropriate for a particular visit. As multiple key informants mentioned, having trusting relationships with one's clinicians is at the root of all quality care, whether conducted in person or virtually. Without that trust, individuals may be unlikely to engage fully in telehealth appointments.

### Implications for health equity

There are many reasons why people are unable to use or prefer not to use telehealth for their appointments. As demonstrated above, these reasons could stem not only from the individual context (such as preference for in-person appointments, or not understanding how to use the technology), but also from the organizational or policy levels (not having access to broadband, service is not appropriate for the patient visit type, the service was not offered to the patient, or provider does not like to use the service due to lower reimbursement rates). It is clear that telehealth is not appropriate in all contexts. The equity concern arises when the appointment could theoretically be conducted in either format and individuals who engage in telehealth are getting equivalent care in a more timely, and/or less expensive manner. Understanding how the different dimensions of patient access are influenced at different socioecological levels^40^ is the first step in developing appropriate, equitable approaches to care.

### Limitations and strengths

There are several limitations to this study. First, while our survey sampling frame was a random sample of patients within the health care system, those who responded had an average age of 61, which will affect the generalizability of the findings. In addition, this study took place in one region of central New York in the middle of the COVID pandemic. The survey was disseminated in August of 2021, when both the community and health care environments were experiencing considerable pandemic-related stressors. Survey responses may be a reflection of some of the general uncertainty during this time.

Second, the survey itself did not undergo a formal validation process. When reviewing survey responses, it was evident that some items were confusing or unclear to respondents. While these items were not included in the analyses, there may have been additional items for which responses could not be considered valid.

Finally, there is a risk of bias in developing themes from a potentially nonrepresentative sample. While we made sure that we interviewed individuals who had varying experiences with telehealth, it is possible we did not capture the full range of experiences. Despite these limitations, by using a conceptual framework, we were able to explore the concept of access as it pertains to telehealth use. Access is a dynamic, multifaceted concept that is influenced by individual-, organization-, and systemic-level factors.

### Recommendations for future research

While the telehealth research canon is full of small pilot projects testing the efficacy of various telehealth interventions,^41^ the pandemic was the first time in history when telehealth was broadly implemented. This analysis highlighted the different dimensions of access that influence telehealth adoption, but future work must explore each of these dimensions in more detail, including developing methodology to assess the individual contribution of each dimension on telehealth use. Future work could also focus on the implementation of various approaches to improve each dimension of access. This work would likely involve community-developed strategies to change the culture around telehealth and work toward instilling confidence in the technology for telehealth-naive individuals.

## Conclusions

Our study made clear that it is necessary to look beyond patient determinants and examine different dimensions of access to better facilitate implementation and sustainment of telehealth. Education for both clinicians and patients, clarifying processes, and building trusting relationships are essential for improving utilization of telehealth.

## Supplementary Material

Supplemental data

## Abbreviations Used

NIHCM: National Institute of Health Care Management Foundation
OR: odds ratio
RUCA: Rural-Urban Commuting Area
SD: standard deviation
SE: standard error
