# Supplementary material for: Exploring Multiple Dimensions of Access to and Preferences for Telehealth Use
Source: Telemed Rep. 2023 Dec 8;4(1):348–58. doi: 10.1089/tmr.2023.0049 (PMC10719637; doi:10.1089/tmr.2023.0049)
Supplement: Supplemental data [file Suppl_Data.docx]

**Use of Virtual Health Care Services Survey**

1. Are you a caregiver completing this survey on behalf of the person addressed on this letter?

Yes

No

1. Is English your first language?

Yes

No

1. In terms of your level of comfort in using different types of technology, are you:

Very uncomfortable

Somewhat uncomfortable

Somewhat comfortable

Very comfortable

1. Do you have any type of personal computer (including laptops) in your home?

Yes

No

1. Do you ever go online to access the internet or send/receive email?

Yes

Yes, but I require help

No

1. Do you have any type of internet access at home?

Yes

No **(skip to question 9)**

1. What devices do you use to access the internet in your home? (select all that apply)

Computer (including a laptop)

Tablet

Cell phone

1. What kind of internet connection do you have at your home?

Dial-up

Broadband (“high speed”)

Satellite

Don’t know

Other (please specify)____________

1. What are the reasons you do not have internet access at home? (select all that apply)

This question does not apply to me: I have internet at home

Don’t know how to use it

Use it at work, school, or library/community center

Don’t need it

Don’t want it

Not available

Not a computer person

Don’t really know about computers

Don’t want the children to use it

Cost/too expensive

Other (please specify)____________________

1. If you do not have internet at home, where do you go for your internet access?

(select all that apply)

This question does not apply to me: I have internet at home

This question does not apply to me: I don’t use the internet

School

Work

Friend or family’s home

Library or other community center

Other_______________

1. Do you own a smartphone (iPhone, Android, etc.)?

Yes

No

1. In the **past month**, how often have you used an internet video-conferencing platform (for example, Zoom, FaceTime, or Google Hangouts)?

Never

Occasionally

Sometimes

Extensively

1. **Prior to COVID** (March 2020), how often did you use an internet video-conferencing platform (for example, Zoom, FaceTime, or Google Hangouts?)

Never

Occasionally

Sometimes

Extensively

1. If you **first used** a video-conference platform **during COVID**, how did learn to use it?

This question does not apply to me

I figured it out on my own

Friend or family member

Workplace provided training

Community center (Office for the Aging, library, etc.) provided training

Religious center provided training

Other (please specify)__________________

1. What were your reasons for using a video conferencing platform (such as Zoom, FaceTime, Google Hangouts) **during COVID?** (select all the apply)

This question does not apply to me: I don’t use any video-conference platform

Staying in touch with family and friends

Work

Attending group meetings (such as school board, Rotary, book club, etc.)

Attending religious services

Receiving health care

Other (please specify) ____________________

1. Have you **ever** had a healthcare appointment that was **not** done in person (where you were not in the clinic/doctor’s office)?

Yes, using a regular telephone

Yes, using a computer, tablet or smartphone

No **(skip to question 18)**

Not sure **(skip to question 18)**

1. Did you have this type of visit **prior to COVID** (March 2020)

Yes

No

Not sure

1. Have you **ever** had a healthcare appointment where you went to the clinic but the doctor was on a computer screen (not physically present in the same room)

Yes

No **(Skip to question 22)**

Not sure **(Skip to question 22)**

1. Was this type of visit with a specialist?

Yes

No **(skip to question 21)**

Not sure **(skip to question 21)**

1. If yes, what specialty (select all that apply)

Cardiology

Dermatology

Endocrinology

Orthopedics

Pulmonology

Psychiatry

Other (please specify)_________________

1. Did you have this type of visit **prior to COVID** (March 2020)?

Yes

No

Not sure

1. Do you have access to the patient portal (also known as MyChart)?

Yes

No **(skip to question 25)**

Not sure **(skip to question 25)**

1. How often have you used the patient portal in the **past 12 months**?

Never

Occasionally

Sometimes

Extensively

1. **Prior to COVID** (March 2020), how often did you use the patient portal?

Never

Occasionally

Sometimes

Extensively

1. For what purpose(s) do you use the patient portal? (select all that apply)

This question does not apply to me: I don’t use the patient portal

Check test results

Send a message to my doctor or nurse

Make an appointment

Request a refill

Receive same day online health care through e-visits

Pay my medical bills

Check my child’s records

Pre-register for an upcoming appointment

View visit notes

Other (please specify)_____________________

1. Would you be willing to travel to a **library or other community center** to use the internet for: (select all that apply)

Emailing friends/family

Making purchases online (books, music, electronics, travel, etc.)

Video-conferencing with my doctor for a medical appointment (private room would be available)

Attending a religious service

Attending a meeting

Attending a support group

None of the above

1. Would you be willing to travel to a **friend or family member’s home** to use the internet for: (select all that apply)

Emailing friends/family

Making purchases online (books, music, electronics, travel, etc.)

Video-conferencing with my doctor for a medical appointment (private room would be available)

Attending a meeting

Attending a religious service

Attending a support group

None of the above

1. What are the **benefits** to remote doctor’s appointments and other health-related programs? (select all that apply)

I don’t have to leave home

I don’t have to wait

I don’t have to miss work

I don’t have to drive

I don’t have to be around other people

Takes less time

More flexibility in choice of appointment times

More flexibility in choice of doctor or nurse

Other (please specify)_________

There are no benefits

1. What are the **barriers** to remote doctor’s appointments and other health-related programs? (select all that apply)

Having to share information over the internet

It’s not in person

Technology is not reliable

I’m not able to use the technology

I don’t want to use the technology

Other (please specify)_________

There are no barriers

1. Given a choice, how would you like to learn about health-related programs and services offered at Bassett? (select only your top choice)

Mail

E-mail

Text messages

MyBassett (patient portal)

Telephone call

1. For a doctor’s visit that could be done either in-person or remotely, which way would you prefer? (select one)

A remote visit, using my computer with video

A remote visit, using a regular telephone

An in-person visit

1. Given a choice of **in-person medical visits,** which option would you prefer?

Office visit

Home visit (doctor comes to your home)

1. Does any disability, handicap, or chronic disease keep you from participating fully in work, school, housework, or other activities?

Yes

No **(skip to question 36)**

1. Does your disability or illness make it difficult to use the internet?

Yes

No

1. Please select all categories that your disability(ies) would fall under

Learning disability

Cognitive/developmental disability

Wheelchair user

Difficulty walking

Difficulty with dexterity/hand use

Totally blind

Partially sighted/low vision

Deaf/non ASL user

Deaf/ASL user

Hard of hearing

Difficulty speaking

Chronic pain

Chronic disease/illness

Mental/emotional disability

Other (please specify)_____________

1. Do you or someone in your household use assistive technology (example: screen reader) to access computers and/or the internet?

Yes

No

Not sure

1. What is your gender?

Male

Female

Non-binary

1. What is your age? ____________________
2. What is your race/ethnicity (select all that apply)

African American or Black

Asian/Pacific Islander

Native American or American Indian

Hispanic or Latino

White

Other_________________

1. What is your annual household income?

Less than $30,000

$30,000-$75,000

More than $75,000

Not sure

1. How hard is it to pay for the very basics like food, housing, heating, medical care, and medications?

Not hard at all

Somewhat hard

Very hard

1. What is your job status? (select all that apply)

Full time employed

Part time employed

Full time student

Part time student

Not currently employed, looking for work

Not currently employed, not looking for work

Unable to work

Retired

1. What is your marital status?

Single, never married

Married or living in a marital like relationship

Widowed

Divorced

Separated

1. Who currently lives with you? (select all that apply)

I live alone

Spouse or partner

Children younger than 18

Children 18 or over

Parent(s)

Other relative(s)

Friend/roommate

1. What is your level of education?

High school degree or less

Some college

College degree or more

1. Do you have health insurance provided through Medicaid?

Yes

No

Don’t know

1. Do you have health insurance provided through Medicare?

Yes

No

Don’t know

1. Do you have a primary care provider (a regular doctor that you see)?

Yes

No

1. In general would you say your health is (select one)

Excellent

Very good

Good

Fair

Poor

1. How long have you lived at your current address?

Under 1 year

1-5 years

6-10 years

11-20 years

More than 20 years

1. Physical Address (Street, City, Zip Code)

_______________________

1. Is there anything else you would share about internet use or access to health care?

Thank you! Please return survey in the enclosed business reply envelope
